# Health Literacy, Misinformation, Self-Perceived Risk and Fear, and Preventive Measures Related to COVID-19 in Spanish University Students

**DOI:** 10.3390/ijerph192215370

**Published:** 2022-11-21

**Authors:** Pilar Bas-Sarmiento, María José Lamas-Toranzo, Martina Fernández-Gutiérrez, Miriam Poza-Méndez

**Affiliations:** 1 Department of Nursing and Physiotherapy, University of Cadiz, University Institute of Research in Social Sustainable Development (INDESS), Institute of Research and Innovation in Biomedical Sciences of the Province of Cadiz, INiBICA, 11009 Cadiz, Spain; 2 Andalusian Health System, 11207 Cadiz, Spain; 3 Department of Nursing and Physiotherapy, University of Cadiz, University Institute of Research in Social Sustainable Development (INDESS), 11009 Cadiz, Spain

**Keywords:** COVID-19, health literacy, behaviors, attitude, surveys and questionnaires, students

## Abstract

The “infodemic” is one of the main obstacles in the fight against the COVID-19 pandemic. In order to face it, health literacy (HL) is essential since it allows for knowledge about COVID-19 and the practice of preventive measures to be fostered. This is especially relevant in university students due to their idiosyncrasy. This study aims to evaluate the level of HL related to COVID-19 (HLC), risk perception, misinformation, and the attitudes and behaviors adopted to prevent the spread of coronavirus in Spanish university students. An online questionnaire was administered to 499 Spanish university students. The HLC index presented a mean of 33.89 out of 50; a total of 63.8% had an inadequate level of HLC. They practiced a mean of 7.54 out of 9 preventive behaviors, and the mean knowledge score was 10.40 out of 13. The HLC showed significantly different scores for the degree, the practice of preventive measures, and some sources of information. The level of HL correlates with the adoption of preventive measures. The higher the severity and perceived susceptibility, the more preventive measures are taken by the students. Therefore, there is a need to strengthen the HL skills of university students and address the dissemination of misinformation. Although caution should be taken when generalizing these results due to the limitations inherent within a cross-sectional study and the convenience sampling, our results can guide the establishment of health education strategies and policies for the management of the infodemic in pandemic situations, according to this target population.

## 1. Introduction

The COVID-19 pandemic has been a significant global public health challenge. The causative agent, SARS-CoV-2, which presents as severe viral pneumonia with a high case fatality rate, has caused a total of 605 million cases and 6.4 million deaths as of 11 September 2022, according to the World Health Organization (WHO) [1].

As already mentioned in previous studies, the COVID-19 pandemic has been accompanied by an explosion of inaccurate information about the disease, making it difficult for the general public to make informed decisions [2].

The “infodemic” (information epidemic), which means too much information, including false or misleading information in digital and physical environments during a disease outbreak, has been the main obstacle in the fight against this pandemic [2,3,4,5,6]. The WHO termed the COVID-19 misinformation situation as an ‘infodemic’ swarming with conspiracy theories, propaganda, and unproven scientific claims regarding the diagnosis, treatment, and prevention of the disease [7,8], which has made reliable information more difficult to find and discern, and has allowed rumors to spread more quickly, putting public health at risk by hindering the implementation of effective preventive measures [8]. Health Literacy (HL) is essential to address this [9,10] since there are studies that identify that people with low HL have been found to show lower awareness, knowledge, and protective behavior, which might result in a greater risk of COVID-19 infection [11,12,13,14,15,16]. An adequate level of HL facilitates the identification of reliable information on COVID-19, improves knowledge, and enables the practice of protective health behaviors [17]. Some studies identify HL as a “social vaccine” to face the COVID-19 infodemic [10,11,18].

The problem of the “infodemic” is especially critical for university students because of the frequent use of social networks and the internet [19,20,21], where information is rapidly disseminated and where a large amount of false or misinformation circulates [22,23,24,25,26]. They are also the most active online, interacting daily with an average number of 5 digital platforms (such as Twitter, TikTok, WeChat, and Instagram). In order to better understand how they are engaging with technology during this global communication crisis, an international study was conducted covering approximately 23,500 respondents (Generation Z and Millennials) in 24 countries across five continents [27]. This study concluded that the challenge is in recruiting fake news and actively countering it rather than ignoring it. Similarly, university students are a relevant population group regarding health behaviors and risk perception. They are more likely to engage in risky behaviors due to their particular characteristics, such as being young people, their socialization in groups, and feeling invulnerable. Although university students are less likely to become ill, adherence to preventive behaviors is critical in this group as they can spread the disease to the population due to person-to-person transmission [28]. We cannot forget that health behaviors and the risk perception of a population in the face of an epidemic are essential when authorities design an action strategy. Indeed, the WHO is keenly aware of how important meaningful youth engagement is to the COVID-19 pandemic. They have an important role to play both in terms of helping to reduce transmission and in engaging in the response [29].

**Figure 1:**
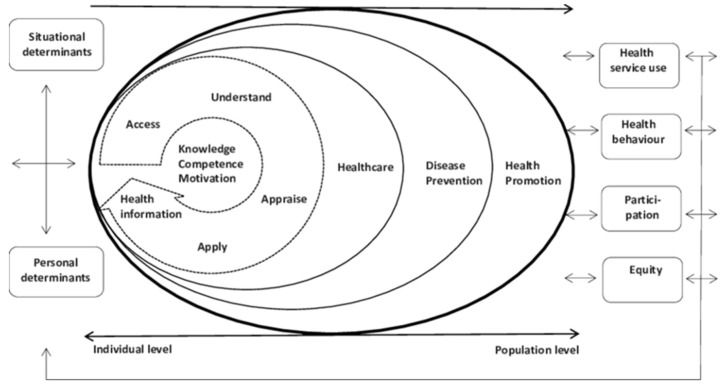
Integrated HLS-EU model of Health Literacy [39]. The frameworks associated with the three domains represent a progression from an individual towards a population level.

It has been emphasized that HL plays an important role in pandemic control, and the need to take it into consideration in public health messages to reach everybody in the fight against the virus [2,30,31,32]. In fact, the COVID-19 pandemic has highlighted that poor HL is a globally underestimated public health problem [9]. In addition, studies to date suggest a positive association between HL, COVID-19-related knowledge, and preventive behaviors [14,17,33,34,35,36,37,38]. People have difficulty evaluating the reliability of the information on COVID-19, and a population with limited HL is more likely to feel confused due to a large amount of information available in the media and the internet [2]. In the European context, HL has been defined as “the knowledge, motivation and competencies to access, understand, appraise, and apply information, to make judgments and decisions in terms of healthcare, disease prevention and healthy behaviors, to maintain and promote quality of life throughout the life course” [39]. This definition is based on a conceptual model (Figure 1) that combines four dimensions referring to health information processing (the competencies related to the process of accessing, understanding, appraising, and applying health-related information) with three levels of domains (healthcare, disease prevention, and health promotion) that yield a matrix with 12 dimensions of health [39]. There is evidence that HL predicted health-protective behaviors in university students [12,34,40,41,42]. In addition, HL is context-dependent. This means people with generally acceptable HL skills can still face HL challenges in some contexts, such as a pandemic. Therefore, regarding the infodemic, a comprehensive study into the HL levels of the target populations to determine the best strategies to improve it should be conducted [43].

On the other hand, according to the health belief model [44] (one of the most used theoretic frameworks in health psychology to explain health and preventive behaviors), behaviors are the result of a set of beliefs and internal valuations that an individual uses in a particular situation. The desire to avoid illness (or, if ill, to get healthy) and the belief that a specific healthy behavior can prevent illness (or if ill, the belief that a specific healthy behavior can increase the chance of regaining health) would be the basis of the preventive behavior. Thereby, perceived susceptibility, perceived severity, perceived benefits, and perceived barriers are the dimensions of the health belief model [45]. The perceived susceptibility to a certain health problem fundamentally refers to the subjective perception that each human being has about the risk of falling ill. The perceived severity refers to beliefs about the seriousness of contracting a certain disease or leaving it untreated once contracted. Studies show that perceived susceptibility and perceived severity are significant determinants of health behaviors [46,47,48,49,50] and therefore affect the adoption of preventive behaviors. The perceived benefits are considered as the relative effectiveness that the different behaviors available in their repertoire may have when dealing with the disease; various studies show that perceived benefit can be a predictor variable of preventive behavior [51,52,53,54,55]. And the perceived barriers are those that oppose the execution of the behavior by the individual; different studies revealed that perceived barriers have a significant negative impact on COVID-19 preventive behavior [46,55,56,57,58]. Therefore, understanding the risk perception of the university population and the sources of information that they trust is essential for allowing effective communication. Similarly, optimal HL in academic settings enables people to use reliable health information and empowers them to adopt preventive behaviors, helping to curb the transmission of SARS-CoV-2 by guiding idiosyncratic policies [29].

However, despite the efforts made to assess COVID-19-related HL, there is little evidence of its determinants. There are population-based studies in different countries, such as the ones carried out in Spain [59], Germany [60], Turkey [61], and Canada [62]; some studies have analyzed the knowledge and attitudes of students from Indonesia [63], Denmark [18], Pakistan [40,64], Egypt [65], and Jordan [66]. However, to our knowledge, no study has examined the association between HL and COVID-19 preventive behaviors in Spanish university students.

Therefore, this study aims to evaluate the level of HL related to COVID-19 (HLC), the risk perception, the misinformation, and the attitudes and behaviors adopted to prevent the spread of coronavirus in Spanish university students.

## 2. Materials and Methods 

### 2.1. Desing and Sampling

A cross-sectional descriptive study. The sample consisted of 499 Spanish university students through a nonprobabilistic convenience sample.

The inclusion criteria were students who are currently enrolled in a university degree, master’s degree, or doctorate in Spain and who voluntarily want to participate.

The university population was invited to answer an online “Google Forms” questionnaire distributed via WhatsApp and social networks.

### 2.2. Variables and Instruments

The questionnaire used in the COSMO Spain study [61], the health literacy questionnaire related COVID-19 (HLCQ), was adapted and prepared for online distribution. It was based on the survey tool developed by the Regional Office for Europe of the WHO [59] and assessed the participants’ self-perception. 

The initial questionnaire was sent to 5 people as a pilot before the link was sent to participants. It consists of 9 sections:

Sociodemographic characteristics: The sociodemographic variables collected were age, gender, size of the population of residence (<2000; 2000–50,000; 50,000–400,000; >400,000), degree, and current academic year.

Health Literacy Questionnaire related to COVID-19: HLCQ was adapted from the items included in the COSMO-WHO [67,68]. The questionnaire items, originally in English, were translated by professional translators, reviewed, validated, and slightly modified by the COSMO-Spain team [59,69]. The scale showed a Cronbachߣs alpha of 0.87, and the item total corrected correlation was 0.49–0.68 [69]. The questionnaire, which assessed citizens’ perceived difficulty in accessing, understanding, evaluating, and applying health information follows the structure of the European health literacy survey questionnaire (HLS-EU-Q) [70]. In the present study, a 9-item version was used. As can be seen in Supplementary Materials S1, the instrument begins with a general question followed by specific statements so that participants rate their perceived difficulty on a four-point scale: very difficult, difficult, easy, and very easy. 

In accordance with the original questionnaire, the HLCQ index was standardized from 0 to 50 [index = (mean − 1) × (50/3)] using the mean of all items for each respondent. HLCQ scores are classified as “inadequate” (0–25), “problematic” (26–33), or “sufficient” (34–50 points) [17,71,72,73].

Information sources: The frequency and sources of information to obtain information about COVID-19 were assessed. The primary sources of information that were included were the news, radio and TV talk shows, press conferences, national press, social media, the internet, the Ministry of Health, the Consumer Affairs and Social Welfare website (MHCSW), and the WHO. The frequency of use of each of these was assessed on a scale from 1 (never) to 5 (very often).

Confusion about the veracity of information: To assess the confusion about the veracity of the information, the participants were asked if they had received any information/news about COVID-19 that they had had difficulty distinguishing as to whether it was true or false.

Knowledge about COVID-19: Knowledge was assessed by 13 items about the transmission and prevention of COVID-19. The first 6 questions include the possible transmission routes: droplets when coughing/talking (right), contact with contaminated surfaces (fake), physical contact with someone infected (right), blood transfusion (fake), insect bite (fake), and contact with pets (fake). They were also asked whether they believed that people without a fever can be contagious. The last 6 items focused on the beliefs of university students about the correct use of masks: washing hands before and after use, whether it should cover the nose and mouth, whether it should be touched only by the ear band, whether it should be removed for coughing or sneezing, and whether its function is to avoid infecting others and to protect oneself from being infected.

Risk perception: Taking into account the health belief model to evaluate perceived risk related to COVID-19, 3 questions were asked: (a) perceived severity: “How severe do you think the disease would be if you were infected with coronavirus?”. Possible responses for this item are “very mild, mild”, “intermediate”, or “severe, very severe”; (b) perceived susceptibility: “How likely do you think you are to get the coronavirus?” Responses range from 1 (“not at all likely”) to 5 (“very likely”), and (c) perceived invulnerability: “As things currently stand, managing to avoid getting infected with the coronavirus is...”. The response is on a Likert scale from 1 to 5, with 1 being “very difficult” and 5 being “very easy”.

Preventive measures: Preventive measures included in the questionnaire were washing hands with soap and water frequently, using hand sanitizer or other disinfectants, not going to social/family gatherings, staying at home in case of symptoms, keeping a physical distance (minimum 2 m), using the mask as recommended, disinfecting surfaces, avoiding public transport, not going out of the house, and going out as little as possible. Participants had to select the measures taken in the last 7 days to prevent the transmission of COVID-19.

Fear: The fear feeling produced by COVID-19 was evaluated through 3 questions. The feelings produced by the coronavirus were assessed on a scale of 1 (“fear”) to 5 (“it does not make me afraid”), and the last two questions included concerns, asking how much you are worried about the coronavirus in general (“not at all”, “a little”, “quite a lot”, “a lot” or “very much”) and how much you are worried about from 1 (“not at all”) to 5 (“a lot”) several specific aspects (saturation of health services, their physical or mental health, people who go without a mask or wear it incorrectly, a new confinement and discussions and fights with family members to maintain the rules).

Level of trust in information: The last question was related to students’ trust in information from news programs, radio and TV talk shows, press conferences, national press, health professionals, conversations with family/friends, social networks, the internet, the MHCSW, the WHO, and COVID-19 hotlines. Each of these items was measured on a scale from 1 (“no confidence”) to 5 (“a lot of confidence”).

### 2.3. Data Collection

The data were collected through the online questionnaire “Google Forms” between 28 January and 28 February 2021, during the so-called “third wave” of the COVID-19 pandemic and with most university students in a non-face-to-face mode. During these dates, mobility restrictions and limitations on opening hours and seating capacity in commercial establishments in Spain were maintained. The use of face masks was mandatory for people aged 6 years or older in enclosed spaces and outdoors. The government opened all internal borders among autonomous communities as well as international travel restrictions between other European Union countries and the United Kingdom. Other restrictions related to mass gatherings and the closure of public spaces were handled by each autonomous community independently.

### 2.4. Statistical Analysis

A descriptive analysis was carried out to determine the sample distribution for each of the variables studied: sociodemographic variables, HL related to COVID-19, information sources, confusion over the veracity of information, knowledge about COVID-19, preventive measures, risk perception, fear, and level of trust in information.

Prior to the analysis, the normality of the variables was studied through the Kolmogórov-Smirnov and/or Shapiro–Wilk test, depending on the sample size, and Levene’s test was used to assess the homogeneity of variance. Quantitative variables are expressed in terms of summary (means, modes, and medians) and dispersion (standard deviations and ranges), and categorical variables in frequency and percentages.

According to a study on the normality of the variables, the Mann–Whitney U test and the one-way analysis of variance (Kruskal–Wallis test) were used to compare the mean difference between two groups or more. In the case of linear relationships, the Spearman’s correlation coefficient was calculated. Statistical significance was determined for a value of *p* < 0.05.

The statistical treatment of the data was carried out with the SPSS statistical package, version 23.0.

### 2.5. Ethical Considerations

This work was conducted in accordance with the Declaration of Helsinki [74]. The aim of the study and the anonymity of participants, as well as the voluntary character of their participation were all explained before the participants started answering the questionnaire and before their informed consent was obtained. The participants were informed that the data obtained would be used only for research purposes.

## 3. Results

### 3.1. Sociodemograpfic Profile

The sample consisted of 499 university students, and the age of the students ranged between 18 and 36 years, with a mean age of 21.50 years (SD = 2.86) (Table 1). 

Most of the participants were students from the Health Sciences (49.9%) followed by Social and Legal Sciences (30.5%). On the other hand, the percentage of students in the Sciences (8.4%), Engineering and Architecture (8%), and Arts and Humanities (3.2%) was low. Most students were enrolled in the second and fourth years of their university degrees (55.6%).

### 3.2. Confusion about the Veracity of Information

A total of 78.4% (n = 391) of university students had difficulty distinguishing whether a news item or information about COVID-19 was true or false. If we divide the sample between students from the Health Sciences and those enrolled in other different degrees, the results show that, for the first group, the percentage was 81.5% (n = 203), and for students of the rest of the degrees it was 75.2% (n = 188).

### 3.3. Risk Perception

The majority of university students think that, if they were infected with coronavirus, the illness would be “very mild/mild” (42.1%) or “intermediate” (54.3%). While only 3.6% think it would be “severe, very severe”. These results are the same if we divide the sample between students in the Health Sciences and those who are enrolled in other different degrees. For the first group, 54.2% consider that, if they were infected with coronavirus, the illness would be “intermediate”, and in the case of the nonhealth degrees, it was 54.4%.

The mean score for perceived susceptibility to infection was 3.28 out of 5 points (SD = 0.967), and for perceived invulnerability was 2.62 (SD = 1.007). Likewise, among the students belonging to the Health Sciences, the perceived susceptibility was 3.31 (SD = 0.900), and in the rest of the students, it was 3.26 (SD = 1.031). As for the perceived invulnerability among students belonging to Health Sciences versus those belonging to other degrees, it was 2.54 (SD = 0.996) and 2.70 (SD = 1.149), respectively.

### 3.4. Preventive Measures

Most of the students had carried out almost all of the preventive measures in the last seven days (prior to the questionnaire) to avoid COVID-19 infection; the mean of the total number of preventive measures taken was 7.54 out of 9 points (SD = 1.56).

The measures that were adopted by most of the students were the following. Using a mask as recommended (97%), staying at home if they have symptoms (95.2%), using hand sanitizer or other disinfectants to wash their hands (94.4%), washing hands with soap and water (92.6%), keeping a physical distance (84.6%), avoiding public transport (81.6%), avoiding going out, and going out as little as possible or teleworking (80.4%). On the other hand, the measures least carried out by the students were the following. Avoiding going to social/family gatherings (74.3%) and disinfecting surfaces (54.5%), with the latter being the measure that the lowest number of students carried out. 

Among the different groups according to the university degree being studied, some differences were found; among the students belonging to the Health Sciences, the measures most adopted were the use of a mask as recommended (98.4%), the use of hand sanitizer (96.8%), and staying at home if they had symptoms, and washing hands with soap and water (both with 95.2%); in contrast, among the students from other university degrees, the order was the following. The use of a mask as recommended (95.6%), staying at home if they had symptoms (95.2%), the use of hand sanitizer (92%), and washing hands with soap and water (90%).

### 3.5. Knowledge about COVID-19

The mean score was 10.40 out of 13 points (SD = 1.19). Statistical differences were observed between the groups (z = −2.722; *p* = 0.006) according to the university degrees in which the students were enrolled (Health Science students: 10.55; SD = 1.159 vs. Science students: 10.24; SD = 1.216).

Most of the students had an optimal level of knowledge about COVID-19. However, many had misconceptions about some of the routes of transmission of the coronavirus. In particular, they incorrectly believed that transmission occurred via physical contact with someone infected (87.4%), as well as with contaminated surfaces (68.3%), and blood transfusion (40.3%). The remaining items regarding the transmission routes were correct by more than 90%.

Regarding mask use, the majority had adequate knowledge, with more than 93% of people answering every item correctly. The item that obtained a lower percentage for correct answers (84%) referred to masks being used to protect against infection. 

### 3.6. Health Literacy Related to COVID-19

The mean HLC index score of the total sample was 31.59 (SD = 10.21), which corresponds to a problematic HLC level. A total of 36.3% of the respondents (n = 181) had a sufficient HLC level, followed by those who had a problematic HLC level (n = 165; 33.1%), and the remaining 30.7% (n = 153) had an inadequate level of HLC. In this case, the mean HLC index score of the Health Sciences students ($\overset{-}{x}$ = 32.72; SD = 9.73) was significantly higher (z = −2.215; *p* = 0.027) than that of the other degree programs ($\overset{-}{x}$ = 30.46; SD = 10.55). 

Participants found the following items challenging to understand: what authorities say about the coronavirus, understanding restrictions, and recommendations, assessing whether media information about the coronavirus is reliable, assessing when they need to go to the doctor for a problem unrelated to the coronavirus, and learning about restrictions related to the coronavirus. The mean scores for these statements ranged from 2.24 to 2.84.

### 3.7. Correlation between HLC and Other Variables

Concerning the sociodemographic characteristics, no significant correlation was found between HLC and age (r_s_ = 0.02; *p* < 0.69). No significant differences were observed for sex (z = −1.55; *p* = 0.12), size of the population in which they reside (X^2^_KW_ = 2.27; *p* = 0.52), course (X^2^_KW_ = 7.32; *p* = 0.12), confusion in the veracity of information (z = −1.59; *p* = 0.11), or with perceived severity (X^2^_KW_ = 2.63; *p* = 0.27). Although there were no significant differences between the HLC index and gender, the level of inadequate HLC was significantly more frequent in women (z = −2.24; *p* = 0.02). The results of the HLC index were significantly different according to university degree (X^2^_KW_ = 12.09; *p* = 0.02). The mean HLC index was higher for the Sciences ($\overset{-}{x}$ = 33.65; SD = 10.39), Health Sciences ($\overset{-}{x}$ = 32.72; SD = 9.73), and Engineering and Architecture ($\overset{-}{x}$ = 32.55; SD = 8.45). Whereas for the Arts and Humanities” ($\overset{-}{x}$ = 30.72; SD = 11.83) and the Social and Legal Sciences ($\overset{-}{x}$ = 29; SD = 10.77), it was lower (Table 2).

HL was not significantly correlated with perceived susceptibility, invulnerability, or consulting information on social media and talk shows (Table 3). In contrast, there was a significant correlation with frequently consulting information from television news (r_s_ = 0.097; *p* = 0.003), press conferences (r_s_ = 0.17; *p* < 0.001), national press (r_s_ = 0.17; *p* < 0.001), the Internet (r_s_ = 0.13; *p* = 0.003), the website of the MHCSW (r_s_ = 0.29; *p* < 0.001) and the WHO (r_s_ = 0.23; *p* < 0.001) (Table 3). Students who consulted information about COVID-19 on the Internet, the MSCBS website, and the WHO more frequently had a higher mean HLC index (33.11 vs. 36.39 vs. 35.76) than those who consulted these sources less frequently (27.71 vs. 28 vs. 28.72). 

Regarding the different preventive measures, there were significant differences in the HLC index according to the following measures (Table 2): not going to social or family gatherings (z = −4.17; *p* < 0.001), keeping a physical distance (z = −2.42; *p* = 0.01), using the mask following the recommendations (z = −3.36; *p* = 0.001), not going out of the house, and going out as little as possible or teleworking (z = −2.79; *p* = 0.005). Students who carried out these measures had a higher HLC index.

### 3.8. Correlation between the Preventive Measures Taken and the Different Variables

The total preventive measures were significantly associated (X^2^_KW_ = 22.16; *p* < 0.001) with university degrees. Health Science and Science students were the ones who practiced the most preventive measures ($\overset{-}{x}$ = 7.83 vs. $\overset{-}{x}$ = 7.45), and Engineering and Architecture students were the ones who adopted the least preventive measures ($\overset{-}{x}$ = 6.77).

Adopting preventive measures was significantly correlated with fear of COVID-19 (r_s_ = −2.83; *p* < 0.001). The more fearful they were of the disease, the more preventive measures they took. The mean number of preventive measures for the highest level of fear was 8.22; for the lowest, it was 6.15 on average. Even though during the third wave of the pandemic, the preventive measure of disinfecting surfaces was dismissed, we observed a statistically significant relationship between fear of COVID-19 and the use of these measures (z = −4.174; *p* < 0.001). Similarly, a statistically significant relationship was found between feelings of concern and the implementation of this preventive measure (z = −5.267; *p* < 0.001).

As for the relationship between preventive measures and perceived risks, it is significantly related to the perceived severity of contagion (X^2^_KW_ = 6.72; *p* = 0.03), and a significant correlation is observed with perceived susceptibility (r_s_ = 0.15; *p* = 0.001), but no significant correlation is found with perceived invulnerability (r_s_ = −0.04; *p* = 0.38). The higher the severity and perceived susceptibility, the more preventive measures students will take. Specifically, university students who consider the illness mild or very mild took an average of 7.32 out of 9 preventive measures, compared to an average of 7.75 for those who consider the illness severe or very severe. The average number of preventive measures taken by students who think they are not at all likely to catch the disease was 7, while the average number of preventive measures taken by those who thought it was very likely to catch the disease was 7.8. 

### 3.9. Correlation between Knowledge and the Different Variables

The level of knowledge related to COVID-19 was significantly associated with washing hands with soap and water (z = −2.88; *p* = 0.004) and not going to social gatherings (z = −2.51; *p* = 0.012). Students who reported washing their hands with soap and water achieved a higher level of knowledge ($\overset{-}{x}$ = 10.45) than those who did not wash their hands ($\overset{-}{x}$ = 9.78); additionally, those who did not go to social gatherings had a higher score ($\overset{-}{x}$ = 10.498) than those who did ($\overset{-}{x}$ = 10.12).

Finally, no significant correlation could be established between the level of knowledge and the total number of preventive measures adopted or the HLC index. However, we did find a correlation between the HLC index and the total number of preventive measures taken (r_s_ = 0.15; *p* = 0.001). The higher the HLC index, the more preventive measures were practiced. University students who took only three preventive measures had a mean HLC of 21.76, while those who took all measures had a mean HLC of 33.13.

## 4. Discussion

To our knowledge, few studies have investigated COVID-19-related HL and the adoption of preventive measures in university students. This study aimed to evaluate the level of HL related to COVID-19 (HLC), the risk perception, the misinformation, and the attitudes and behaviors adopted to prevent the spread of the coronavirus among Spanish university students. The results showed that a high number of preventive measures were taken (7.54 out of 9), and the level of knowledge was moderate (7.54 out of 10) among the participants. On the other hand, the HL regarding COVID-19 among university students was not adequate, as the mean HLC index (31.59) did not reach the level of HLC “sufficient”. This result is similar to the one obtained in the COSMO-Spain project with a representative sample of the Spanish population [59], where the mean index score was 33.89, and more than 60% did not have adequate (problematic or inadequate) levels of HL, akin to the 63.8% in the present study. These findings also seem to agree with the results of the study by Yuan et al. [73], who reported that 45.9% of university students did not have adequate levels of HL. Furthermore, Okan et al. [17] found that, in the general population in Germany, just over half of the sample had inadequate COVID-19-related HL. In all three of the studies mentioned, the level of HL is higher than the score in our study.

However, in the study by Shaukat & Naveed [40], the proportion of university students with inadequate HLC levels was even higher than in the present study, as they found that 81.55% of university students had low to moderate levels of HL related to COVID-19.

These findings were quite surprising and worrying because if university students belonging to a population group with higher education do not have sufficient HL, we should ask ourselves whether individuals with less education, the illiterate, and the rural population might have even lower levels of HLC. On the other hand, we are also concerned about the consequences for a population that will be more prone to risk-taking. Inadequate HL among university students may be due to inappropriate sources of information. The most commonly used sources among the participants are the internet and social networks, with the attendant risk of finding unreliable information [22,23,24,25,26]. Moreover, more official and, therefore, more reliable sources, such as the MSCBS website and the WHO, were the least consulted. Students who frequently consulted the MSCBS website and the WHO for information about COVID-19 had a higher HLC score. Similarly, in a study by Rosário et al. [74], it was found that the frequent use of public agency websites increased the likelihood of sufficient digital health literacy.

However, it is also true that they had little trust in social media and the internet and relied more on information from health workers, the MSCBS, and the WHO. Nonetheless, 78.4% of the university students had difficulty distinguishing whether a news item or information piece about COVID-19 was true or false, presenting higher results than those obtained in other studies [18,75,76]. Okan et al. [17] found that 56% of the general German population was confused about COVID-19 information, showing more confusion among university students than in the present study. This may be due to university students’ frequent use of unreliable sources and their low trust in them.

The difficulty for a high percentage of the sample to identify misinformation about COVID-19 indicates the need to support students by providing high-quality information that allows them to assess the reliability of the source and counter misinformation about COVID-19 on social media.

The mean HLC scores show that the biggest challenges for the students were understanding what the authorities said about coronavirus, understanding the restrictions and recommendations, assessing whether the information given by the media about coronavirus was reliable, assessing when they needed to go to a doctor for a problem unrelated to coronavirus, and learning about the restrictions related to COVID-19. These difficulties coincided with those obtained in round 4 (25 January–1 February 2021) of the COSMO-Spain project [59].

The majority of students (61.3%) found it difficult or very difficult to assess whether the information about COVID-19 provided by the media was reliable, obtaining higher results than the ones in the studies by Okan [17] and Dadaczynski et al. [75], where 47.8% and 42.3% of respondents, respectively, reported that it was difficult or complicated to as-sess the reliability of the information provided by the media. When students access misinformation or false information and have difficulty making judgments about the veracity of the information, they are unlikely to identify that information as “fake news”, and this may prevent engagement in effective health behaviors. These findings suggest the need to implement health education measures to strengthen students’ HL skills [77]. 

Age and the HLC index did not correlate significantly in our study, as the sample has a specific age range with a minimal margin. The association with the rest of the sociodemographic data was not significant, except for with university degree. The HLC index was higher in the Health Sciences, Sciences, and Engineering and Architecture degrees. In the Social and Legal Sciences and Arts and Humanities, more emphasis should be placed on health education. Although there was no significant association between the HLC index and gender, the “inadequate” HL level was significantly more frequent in women (70%). These results can be explained by the attendance of mostly women in the sample.

Students with higher levels of HLC took more preventive measures. The same correlation was found in other studies [13,16,73,78,79,80], where the higher the level of health literacy, the more preventive behaviors were carried out.

In general, students reported adequate prevention measures. Those who did not attend social or family gatherings, kept their distance, wore masks as recommended, and left the house as little as possible had a higher index of HLC.

The results of the study by Falcón et al. [69] compared to the results of the present study showed a lower frequency in the adoption of preventive measures in participants from the general Spanish population. This difference stands out for measures such as staying at home if presenting symptoms (32.6% compared to 95.2% in the present study), avoiding public transport (35.8% compared to 81.6%), and not attending social gatherings (56.8% compared to 74.3%). In other words, it seems that university students are more aware of adopting preventive measures than the general Spanish population. This difference can also be explained by the fact that the data collection was carried out when the said population was more relaxed about taking precautions against COVID-19 due to the arrival of summer.

The high number of preventive measures taken among the participants could also be explained by the fact that most of them study the Health Sciences; students from this area have more health knowledge, as well as the possible experience of the consequences of the pandemic during clinical practices.

According to Shaukat et al. [34,40], the present study showed that, as fear and concern of COVID-19 increases, the number of preventive measures practiced increases (despite the fact that some preventive measures have become obsolete, e.g., disinfecting surfaces). Regarding risk perception, the higher the susceptibility and perceived severity of the university students (if they were to become infected), the more preventive measures they follow; similar findings have been found in numerous studies [46,47,48,49,50]. Less than 4% of the students considered COVID-19 to be serious or very serious. This was very low compared to the percentage obtained in the fourth round of the COSMO-Spain project (36%) [59]. In contrast, the average score for susceptibility and perceived invulnerability was approximately 3 points out of 5 in both cases. In the case of perceived susceptibility, the results tended more towards being likely to become infected, while invulnerability tended towards the consideration that infection was easy to avoid. The latter could be explained by the aforementioned feeling of invulnerability of young people, the low mortality in this age range, and their knowledge about preventive measures.

Paradoxically, there is no significant correlation between the HLC index and the level of knowledge. This may be because they are all university students, which a highly educated population group, who have been provided with much information about COVID-19 by public administrations and educational centers. There was also no correlation between the level of knowledge and the number of preventive measures taken. However, students who reported washing their hands with soap and water and those who did not attend social gatherings achieved a significantly higher level of knowledge.

Finally, the mean knowledge score was similar to that in Yuan’s study [73]: 14.68 out of 21, or 9.9 out of 13, compared to 10.40 out of 13 in the present study. Despite being a sample consisting of nursing students, they showed less knowledge. This may be because the questionnaire date was almost a year earlier (30 March to 5 April 2020). Regarding mask knowledge, the majority of the participants had adequate knowledge about the usefulness and the correct use of the mask. However, 16% did not know the mask was used to protect them from infection. This was lower than in round 1 (27 July–3 August 2020) of the COSMO-Spain project [59], where 30% were unaware of the usefulness of the mask. Therefore, we can see that the population is becoming more aware of the use of masks over time.

## 5. Limitations and Strengths

This study is not without its limitations. In addition to the limitations inherent to a cross-sectional study, the sample was chosen by nonprobabilistic convenience sampling. A convenience sample can lead to the over-representation of particular groups within the sample. Considering that HLC is context-dependent, that is to say, it may vary in different settings, generalizing the results should be carried out with caution.

In addition, the data collection was conducted during the “third wave” of COVID-19, when limitations and more restrictive measures were maintained due to the spike in cases resulting from the Christmas holidays. After enduring restrictions for long periods and the emergence of contradictory information on COVID-19, the adherence to prevention measures and COVID-19-related HL may have been compromised.

Despite the limitations encountered, our study has strengths to be considered. Thus, it is one of the first studies to measure HLC in university students, providing relevant data on the levels of HLC, risk perception, misinformation, and the attitudes and behaviors adopted to prevent the spread of coronavirus in Spanish university students. Given the difficulty of data collection due to restrictions, a sizeable sample was drawn to overcome the shortcomings of the nonprobabilistic sampling method of convenience.

## 6. Conclusions

The level of HL in the surveyed university students is primarily inadequate and problematic. Despite this, they generally follow preventive measures, having more difficulty keeping social distance, avoiding social/family gatherings, going out as little as possible, and avoiding public transport.

On the other hand, most of the surveyed university students use new technologies to gather information despite their distrust.

The level of knowledge of most of the participants was moderate. They incorrectly believed that physical contact with someone infected, contaminated surfaces, and blood transfusion are all transmission routes. Efforts should be made to reach university students through their usual information channels, increasing the quality of health information available on the internet and social media, and implementing fact-checking strategies on social media, checking that all information is based on scientific evidence. 

Between our participants, the profile of university students who find it challenging to follow preventive measures is that of those who are least afraid of COVID-19 and consider that the disease is not severe and that they are not likely to contract it. The HLC index increases significantly when more preventive measures are taken in total. 

As the scientific literature has demonstrated, infodemia requires strengthening the HL skills of the general population to encourage their adherence to COVID-19 prevention behaviors, particularly with university students. This should include public policy strategies to address the toxic spread of misinformation and misinformation about COVID-19 [9,17]. In addition, university awareness is critical to assist with emergency responses and increase students’ capacity to respond to the HL related to COVID-19. To this end, universities can offer courses on HLC and provide health information to their students and disseminate reliable news about COVID-19 through their websites and social media. 

## Figures and Tables

**Table 1:** Sociodemographic characteristics of the participants.

		N	Mean (SD)
Age		499	21.50 (2.86)
		n (%)	
Sex	Female	384 (77)	
	Male	115 (23)	
Population size of residence	<2000	40 (8)	
	2000–50,000	160 (32.1)	
	50,000–400,000	224 (44.9)	
	>400,000	75 (15)	
University degree	Health Sciences	249 (49.9)	
	Sciences	42 (8.4)	
	Social and Legal Sciences	152 (30.5)	
	Engineering and Architecture	40 (8)	
	Arts and Humanities	16 (3.2)	
Academic course	First	64 (12.8)	
	Second	152 (30.5)	
	Third	106 (21.2)	
	Fourth	125 (25.1)	
	Fifth, Master’s degree, Doctorate	52 (10.4)	

**Table 2:** Categorical variables. Correlations with HLC.

		n (%)	X^2^_KW_ (*p*)	z (*p*)
Sex	Female	115 (23)	-	−1.55 (0.12)
	Male	384 (77)		
Confusion with the veracity of the information	Yes	391 (78.4)	-	−1.59 (0.11)
	No	108 (21.6)		
Perceived gravity	Very mild, mild	210 (42.1)	2.63 (0.27)	-
	Intermediate	271 (54.3)		
	Severe, very severe	18 (3.6)		
Population size	<2000	40 (8)	2.27 (0.52)	-
	2000–50,000	160 (32.1)		
	50,000–400,000	224 (44.9)		
	>400,000	75 (15)		
University degree	Health Sciences	249 (49.9)	12.09 (0.02) *	-
	Sciences	42 (8.4)		
	Social and Legal Sciences	152 (30.5)		
	Engineering and Architecture	40 (8)		
	Arts and Humanities	16 (3.2)		
Academic course	First	64 (12.8)	7.32 (0.12)	-
	Second	152 (30.5)		
	Third	106 (21.2)		
	Fourth	125 (25.1)		
	Fifth, master’s degree, doctorate	52 (10.4)		
Preventive measures	Hand washing	462 (92.6)	−0.44 (0.66)	-
	To use hand sanitizer	471 (94.4)	−1.38 (0.168)	-
	Not going to social or family gatherings	371 (74.3)	−4.17 (<0.001) **	-
	To stay home if you have symptoms	475 (95.2)	−1.92 (0.055)	-
	Keeping a physical distance	422 (84.6)	−2.42 (0.01) *	-
	Using the mask following the recommendations	484 (97)	−3.36 (0.001) **	-
	Disinfect surfaces	272 (54.5)	−0.44 (0.66)	-
	Avoid public transportation	407 (81.6)	−1.07 (0.29)	-
	Not going out of the house, going out as little as possible or teleworking	401 (80.4)	−2.79 (0.005) *	-

* = *p* < 0.05; ** = *p* < 0.01.

**Table 3:** Quantitative variables. Correlations with HLC.

		Mean (SD)	r_s_ (*p*)
Age		21.50 (2.86)	0.02 (0.69)
Perceived susceptibility		3.28 (0.97)	0.00 (0.99)
Perceived invulnerability		2.62 (1.08)	0.07 (0.10)
Frequency of consulting information sources	Television news	3.15 (1.39)	0.097 (0.003) *
	Debate programs	2.27 (1.27)	0.004 (0.93)
	Press conferences	2.42 (1.27)	0.17 (<0.001) **
	National press	2.85 (1.39)	0.17 (<0.001) **
	Social media	3.57(1.36)	0.08 (0.07)
	Internet	3.82(1.19)	0.13 (0.003) *
	MHCSW	2.58 (1.45)	0.29 (<0.001) **
	WHO	2.31 (1.34)	0.23 (<0.001) **
Knowledge		10.40 (1.19)	0.04 (0.34)

* = *p* < 0.05; ** = *p* < 0.01.

## Data Availability

Not applicable.

## Supplementary Materials

The following supporting information can be downloaded at: https://www.mdpi.com/article/10.3390/ijerph192215370/s1. Questionnaire Health Literacy Questionnaire related to COVID-19 (Spanish and English versions).
