# Supplementary material for: Health Literacy, Misinformation, Self-Perceived Risk and Fear, and Preventive Measures Related to COVID-19 in Spanish University Students
Source: Int J Environ Res Public Health. 2022 Nov 21;19(22):15370. doi: 10.3390/ijerph192215370 (PMC9690779; doi:10.3390/ijerph192215370)
Supplement: Supplementary file 1 [file ijerph-19-15370-s001.zip › ijerph-1986374-supplementary.pdf]

Supplementary Material S1.

Health Literacy Questionnaire related to COVID-19 (Spanish version).

¿Cómo de fácil o difícil dirías es para usted…?:

1) …encontrar información sobre los síntomas de COVID-19. [Muy difícil, Difícil, Fácil, Muy fácil]
2) …averiguar qué hacer en caso de sospechar que tiene COVID-19. [Muy difícil, Difícil, Fácil, Muy fácil]
3) …entender lo que dicen las autoridades sobre COVID-19. [Muy difícil, Difícil, Fácil, Muy fácil]
4) …entender las restricciones y recomendaciones que dan las autoridades sobre COVID-19. [Muy difícil, Difícil, Fácil, Muy fácil]
5) …valorar si la información que dan los medios de comunicación sobre COVID-19 es fiable. [Muy difícil, Difícil, Fácil, Muy fácil]
6) …seguir las recomendaciones sobre cómo protegerse de COVID-19. [Muy difícil, Difícil, Fácil, Muy fácil]
7) …decidir cuándo quedarse en casa (y no ir al trabajo/colegio/actividades sociales) y cuándo no. [Muy difícil, Difícil, Fácil, Muy fácil]
8) …valorar cuando necesita ir al médico por un problema sin relación con COVID-19, y cuando no. [Muy difícil, Difícil, Fácil, Muy fácil]
9) …enterarse de las restricciones relacionadas con COVID-19. [Muy difícil, Difícil, Fácil, Muy fácil]

Health Literacy Questionnaire related to COVID-19 (English version).

How easy or difficult would you say it is to…:

1) …find information about symptoms of COVID-19. [Very difficult, Difficult, Easy and Very easy]
2) …find out about political decisions on restrictions related to coronavirus/COVID-19? [Very difficult, Difficult, Easy and Very easy]
3) …find out what to do in case you suspect you have COVID-19. [Very difficult, Difficult, Easy and Very easy]
4) …understand what authorities say about the coronavirus/COVID-19. [Very difficult, Difficult, Easy and Very easy]
5) …understand what authorities say about the coronavirus/COVID-19 restrictions and recommendations. [Very difficult, Difficult, Easy and Very easy]
6) …judge if the information about coronavirus/COVID-19 in the media is reliable. [Very difficult, Difficult, Easy and Very easy]
7) …judge when you need to go to the doctor for non-COVID-19 related problems. [Very difficult, Difficult, Easy and Very easy]
8) …follow the recommendations on how to protect yourself from coronavirus/COVID-19. [Very difficult, Difficult, Easy and Very easy]
9) …decide when to stay at home from social activities/work/school, and when not to. [Very difficult, Difficult, Easy and Very easy]
